# Should the provision of home help services be contained?: Validation of the new preventive care policy in Japan

**DOI:** 10.1186/1472-6963-10-224

**Published:** 2010-08-02

**Authors:** Tomoaki Ishibashi, Naoki Ikegami

**Affiliations:** 1 Department of Health Policy & Management, Keio University School of Medicine, Tokyo, Japan; 2 The Dia Foundation for Research on Ageing Societies, Tokyo, Japan

## Abstract

**Background:**

To maintain the sustainability of public long-term care insurance (LTCI) in Japan, a preventive care policy was introduced in 2006 that seeks to promote active improvement in functional status of elderly people who need only light care. This policy promotes the use of day care services to facilitate functional improvement, and contains the use of home help services that provide instrumental activity of daily living (IADL) support. However, the validity of this approach remains to be demonstrated.

**Methods:**

Subjects comprised 241 people aged 65 years and over who had recently been certified as being eligible for the lightest eligibility level and had began using either home help or day care services between April 2007 and October 2008 in a suburban city of Tokyo. A retrospective cohort study was conducted ending October 2009 to assess changes in the LTCI eligibility level of these subjects. Cox's proportional hazards model was used to calculate the relative risk of declining in function to eligibility Level 4 among users of the respective services.

**Results:**

Multivariate analysis adjusted for factors related to service use demonstrated that the risk of decline in functional status was lower for users of home help services than for users of day care services (HR = 0.55, 95% CI: 0.31-0.98). The same result was obtained when stratified by whether the subject lived with family or not. Furthermore, those who used two or more hours of home help services did not show an increase in risk of decline when compared with those who used less than two hours.

**Conclusions:**

No evidence was obtained to support the effectiveness of the policy of promoting day care services and containing home help services for those requiring light care.

## Background

The public long-term care insurance system (LTCI) established in Japan in 2000 is characterized by broad coverage that includes people with relatively mild disabilities (percentage of those aged ≥65 years who are eligible for such programs: Japan, 16%; Germany, 9.8%; South Korea, 3%) [1]. The threshold for eligibility was set at a relatively low level because many people with mild disabilities were already getting benefits under earlier social programmes and their entitlement had to be respected [2]. As a result, more low-need people than expected applied and became eligible. This rapid growth of enrolees with relatively mild disabilities brought higher than expected total spending [3]. To deal with this situation, a major reform of the system in 2006 focused on sustainability as a key issue, leading to revision of benefits and restrictions of usage for enrolees with mild disabilities (Table 1). Prioritizing benefits toward people with severe disabilities as a response to rising costs has already been observed in Europe and the United States [4-6]. However, rather than explicitly restricting total services for those requiring light care, Japan was unique in introducing a preventive care programme with a goal of reversing and improving their functional status. This programme is at least plausible, in that those in the lightest eligibility level are not very impaired, so that their condition might improve, and there already existed many day care centres which were capable of providing preventive care services.

**Table 1:** Benefits for those in Level 1 (lightest) before and after reform

	Before reform	→	After reform
**Benefit ceiling per** month ($)*	$529		$427

**Drawing and managing care plans**	Agencies selected by users		Local Comprehensive Support Centres operated or supervised by municipalities

**Services available**	Same as other levels (except for institutional services not being available)		Limited to the benefits prescribed for this and the 2^nd ^Level (objective is to prevent progress in disability)

**Home help service**	Same as other levels		Limited to tasks that the user cannot do (cleaning, shopping, cooking etc.)

**Day care services**	Same as other levels		Addition of services for physical strength training, guidance for nutrition and oral function

**Rental equipment**	Same as other levels		Wheelchairs and motor beds not allowed **

* U.S. dollars using purchasing power parity rates (2006).

** Except when judged as needed by the doctor

The new services consisted of the following that were specifically designed to bring functional improvement: physical strength training, and guidance for nutrition and oral function. These services are provided on an individual basis, and not for a group, as is the case for eligibility levels 3 to 7. In addition, the responsibility for care planning and care management for those in the two lightest of the seven eligibility levels has been transferred from agencies selected by the users to Local Comprehensive Support Centres operated or supervised by municipalities [7,8].

The preventive care programme was initiated by the report to the Ministry of Health, Labour and Welfare (henceforth "Ministry") about rehabilitation for elders which stated that "there should be a shift from a 'stroke-based model' to a 'disuse syndrome model' [9]. As an example of 'disuse syndrome', it stated that "despite having the ability to cook and perform other household chores, the use of home help services has led to a gradual decline in ability." The Ministry's Preventive Care Management Manual states that "when selecting services for the two lightest eligibility levels, day care should be actively promoted. By doing so, daily activities and opportunities for social contact would be enhanced so that the disuse syndrome could be prevented or improved. Home help services should be limited to tasks that are difficult to perform independently (cleaning, shopping, cooking etc.), and whether these services are to be provided or not should be considered based on the number of the family members who are living together in the same household, and when community mutual support and other welfare services are not available [8]." Based on the assumption that the preventive care programme would succeed, the Ministry instructed local governments to draw their municipal LTCI plan so that "the percentage of those declining in function from the three lightest eligibility levels to the fourth eligibility level would be reduced by 10% [10]."

The Ministry has not offered sufficient evidence to support this new policy initiative. Although a cohort analysis by the Ministry of 5,000 people eligible for services before and after the LTCI revisions showed a decrease in the rate of who had progressed to heavier eligibility levels after the revisions [11], this study did not consider the length of the observation time before the decline took place, nor was the relationship with service utilization reported. When we reviewed earlier studies that examined the relationship between the use of home help services and functional status, we found none that reported its use led to a decline in function [5,12-14]. We conclude that the validity of the new policy to contain the use of home help services and encourage the use of day care services has not been adequately demonstrated among those using these services.

In the present study, we analysed the relationship between service utilization and eligibility levels from a data set obtained in a joint study with the municipal government of a suburban city of Tokyo. We confined our subjects to those in the lightest eligibility Level 1 in order better demarcate their decline to eligibility level 4 and because there appears to be considerable back and forth between the eligibility levels of 2 and 3 [15]. Although our site had a greater proportion of the population 65 and over than Tokyo, the population 75 and over, in which over 80 percent of those certified as eligible for LTCI is concentrated [15], was less. This is likely to be the main reason why the percentage of the 65 and over who had been certified as eligible is lower than in Tokyo. The percentage of the population 65 and over who were certified in eligibility Level 1 was 1.3% and likewise was lower than in Tokyo. Of those in eligibility Level 1, the percentages of those using services in the lightest level were about the same for the total and for each type of service (Table 2). The sample included all those who had been newly assessed to be in the lightest eligibility level subsequent to the implementation of the new preventive care programme. We also examined care plans to ascertain the level of family support (information which is not available in official datasets). The aim of the present study was to establish the validity of the policy to encourage the use of day care services and to contain the use of home help services by observing decline in functional status as measured by their eligibility level.

**Table 2:** Comparing proportions of elders, those eligible for LTCI, and the use of services in Level 1 for Japan, Tokyo and study site (October, 2008)

	Japan	Tokyo	Study site
**Population**			
Number of people (in thousands)	127,690	12,900	470
Percentage aged 65+	22.1	11.8	18.4
Percentage aged 75+	10.4	9.0	6.8
LTCI eligible*			
Percentage of those age 65+ who are eligible	15.9	14.9	13.8
Percentage for each eligibility level			
Level 1 (lightest)	1.9	2.0	1.3
Level 2	2.2	1.9	2.4
Level 3	2.7	2.3	1.7
Level 4	2.8	2.6	2.6
Level 5	2.5	2.4	2.6
Level 6	2.0	1.9	1.8
Level 7 (heaviest)	1.7	1.7	1.4
Level 1 (lightest)**			
Percentage of those in Level 1 who are receiving benefits	60.0	52.1	50.0
Percentage receiving day care services	39.0	30.5	33.4
Percentage receiving home help services	47.6	62.3	63.7
Percentage receiving rental equipment	14.5	12.5	9.0

* Source: Ministry of Health, Labour and Welfare Website. http://www.mhlw.go.jp/topics/kaigo/osirase/jigyo/m08/0810.html

** Source: Ministry of Health, Labour and Welfare Website. http://www.mhlw.go.jp/toukei/saikin/hw/kaigo/kyufu/2008/11.html

## Methods

### Study design

The sample for this study was drawn from a database of 834 people aged 65 years and over, living in a suburban city of Tokyo, who had applied to LTCI for the first time from April 2007 to October 2008, and had been newly certified as being eligible for Level 1, the lightest eligibility level. The 834 amounted to 88.6% of those currently certified in Level 1 and 1.0% of the population 65 and over in the city. Of the 834, 340 had started using services within three months of certification (many in Japan chose to be just certified so that they could immediately start using services when they wish to do so in the future). From these, the 241 people who had used either day care (102) or home help services (139) -- but excluding the 23 who used both -- were selected for the study. This cohort was observed until October 2009 to track their service utilization, and to see how many were reassessed to eligibility Level 4 or heavier.

Our use of the data was approved by the city after we submitted a formal application for accessing data and explaining the purpose and data to be used.

### Measures

#### Service utilization

LTCI data was used to create time-independent and time-dependent service utilization variables. First, subjects were classified as "day care service users" or "home help service users" based on service utilization within three months from the day of eligibility assessment, and this was defined as a time-independent variable forming a baseline attribute. Next, the number of days for day care services and the number of hours for home help services were added to calculate the cumulative amount of each service utilized during the observation period; these amounts were respectively divided by the number of months of observation in order to create a time-dependent variable of "mean amount of use".

Parenthetically, although records on the hours of services provided are not maintained, we could estimate the amount from the claims data. Payment for LTCI services is set by the Ministry and for home help services for those in the two lightest eligibility levels, there are two forms of bundled fees: a lower fee if less frequent visits are scheduled, generally about once a week, and a higher fee if more frequent services are scheduled, generally about twice a week. Each visit usually lasts 30 to 60 minutes, most of which is used for assistance in instrumental activity of daily living (IADL).

#### Eligibility level

After individuals (or their families) apply to the municipal government, an on-site assessment is conducted of each applicant's physical and mental status. The assessment form contains 79 items, each with a choice of three or four levels, plus space for descriptive statements on particular aspects [3]. These items are analyzed by a government computer program to classify each applicant into one of seven levels (or to reject--about 2 to 3 percent for a new application) [16]. The lightest two levels are for the preventive care services; the other five levels are for the regular LTCI services. An expert committee reviews the classification by taking into account the descriptive statements plus a report from the applicant's doctor. The eligibility decision is then communicated to the applicant within thirty days of applying [17].

In the present study, subjects who were certified as being in the lightest eligibility level were established as the baseline, with a transition to Level 4 or heavier as the measured outcome level. These standards were established for the following reasons. Firstly, subjects in the Level 1 are effectively independent with regard to ADL, despite some degree of IADL disability. On the other hand, ADL disability is clearly found in Level 4 [18]. Therefore, by setting the standard for change in eligibility level at Level 4 or heavier, the change from Level 1 should be interpreted as a change in functional status corresponding to the hierarchical structure of IADL and ADL [19]. The second reason is that the Ministry's goal of a 10 percent reduction in the number of people declining to Level 4 or heavier means that a decline to this level would be an appropriate measure for evaluating the programme's outcomes.

Because people at the two lightest eligibility levels are generally re-evaluated after six months, we obtained the data on eligibility level from the municipality every 6 months commencing in April 2007.

#### Other variables

Data for the baseline socio-demographic variables of sex, age, and economic status (indicated by level of LTCI premiums, which varies by income) were provided by the municipality. Information regarding the relationship of the primary caregiver and time spent providing care were obtained from the care plans drawn up at the start of service utilization.

### Statistical methods

To evaluate demographic differences between the home-help and day-care groups, categorical variables were compared using the chi-square test, and interval variables were compared using the *t*-test.

The relative risk of decline in functional status related to service use was calculated using Cox's proportional hazards model. In addition to an age-adjusted analysis, a multivariate analysis was conducted with covariates of economic status, relationship of primary caregiver, and time spent providing care.

Whether they used either home help or day care services was a time independent variable, and the average hours of home helper service during the observed months was a time dependent variable. In both groups, only newly certified users who had started receiving services within three months of being certified were included in the sample.

## Results

### Baseline characteristics of the participants

Among basic attributes for each of the services, no significant difference was seen in gender or economic status. However, users of day care were nearly five years older than users of home help. By a large margin, the primary caregiver for people going to day care were likely to live in the same household, while home-help users were more likely to have no caregiver or one who came from outside the household (Table 3).

**Table 3:** Cohort characteristics at baseline (N = 241)

	Day care services (n = 102)		Home help services (n = 139)		P value
**Age**, mean ± SD	82.1 ± 6.7		77.5 ± 5.5		0.000
**Gender**					
Male	36	(35.3)	55	(39.6)	0.591
Female	66	(64.7)	84	(60.4)	
Economic status *					
Low	47	(46.1)	71	(51.1)	0.644
Middle	22	(21.6)	24	(17.3)	
High	33	(32.4)	44	(31.7)	
**Caregiver**					
Spouse	27	(29.8)	21	(13.9)	0.000
Other family (same household)	41	(36.0)	21	(15.3)	
Other family (separate household)	30	(28.1)	57	(42.4)	
None	4	(6.1)	30	(28.5)	
**Time when caregiver can provide care**					
24 hours	36	(35.3)	9	(6.5)	0.000
Not during day time working hours	35	(34.3)	21	(15.1)	
Episodic or none	31	(30.4)	109	(78.4)	

* Economic status is classified as low: household tax-exempt, middle: individual tax-exempt but household taxed, high: individual taxed.

### Decline

The mean observation period was 18.0 months (SD ± 7.3). During the observation period, of the 241 people, 67 (27.8%) had declined to eligibility Level 4 or more (excluding those who had died, 2 (0.8%), and those who had moved out, 2 (0.8%)). The proportion declining was greater for users of day care services at 37.3%, when compared the users of home help services at 20.9%.

### Cox's Proportional Hazards Model

The result of the age-adjusted Cox's proportional hazards model was that home help users were roughly half as likely as day care users to decline to eligibility Level 4 or more (Table 4). This finding also held for a multivariate analysis controlling for primary caregiver, hours of care available, and economic status. Similarly, in an analysis stratified by whether or not the primary caregiver lived with the user, the hazard ratio was lower for home help services than for day care services (co-resident: HR = 0.17, 95% CI: 0.05-0.56, p < 0.001, non co-resident: HR = 0.42, 95% CI: 0.14-0.55, p = 0.044).

**Table 4:** Adjusted hazard ratios (HR) of decline (N = 241)

	Percentage declined	Age adjusted		Multivariate adjusted**	
		HR (95% CI*)	**p value**	HR (95% CI*)	**p value**

**Gender**					
Male	31.9	1.0 (Ref.)	-	1.0 (Ref.)	-
Female	25.3	0.81(0.50-1.32)	0.401	0.68(0.38-1.22)	0.195
**Income Level**					
Low	26.3	1.0(Ref.)	-	1.0(Ref.)	-
Middle	30.4	1.06(0.56-2.02)	0.849	1.14(0.58-2.25)	0.694
High	28.6	1.12(0.65-1.93)	0.690	1.02(0.54-1.94)	0.954
**Caregiver**					
Spouse	22.9	1.0 (Ref.)	-	1.0 (Ref.)	-
Other family member (same household)	33.9	1.39(0.65-2.94)	0.393	1.47(0.63-3.45)	0.378
Other family member (separate household)	31.0	1.41(0.70-2.86)	0.333	2.25(0.89-5.65)	0.084
None	18.2	0.88(0.35-2.22)	0.789	1.66(0.52-5.29)	0.396
**Time when caregiver can provide care**					
24 hours	33.3	1.0 (Ref.)	-	1.0 (Ref.)	-
Not during day time working hours	35.7	1.17(0.60-2.28)	0.652	1.08(0.49-2.35)	0.851
Episodic or none	22.9	0.75(0.39-1.43)	0.385	0.67(0.27-1.70)	0.404
**Use of service**					
Day care services	37.3	1.0 (Ref.)	-	1.0 (Ref.)	-
Home help services	20.9	0.54(0.32-0.91)	0.022	0.55(0.31-0.98)	0.043

* Confidence Interval ** Adjusted for all listed variables

Next, the amount of home help services was analyzed to investigate whether there was any dose-response relationship. When those who used two or less hours (170) were compared with those using more (71), the latter did not show any increase in the hazards ratio (< 2 hour per week: HR = 1.0(Ref.), ≧ 2 hour per week: HR = 1.05, 95% CI: 0.51-2.19, p = 0.888). In day care services, when compared with those who had used one day per week or less (187), those using more (54) were associated with a similar risk of endpoint (≦ 1 day per week: HR = 1.0(Ref.), > 1 day per week: HR = 0.92, 95% CI: 0.52-1.62, p = 0.766).

## Discussion

This study focused on the validity of the government policy to contain home help services and encourage day care services. We limited our subjects to those who had actually started using services after the implementation of the reform and to those in the lightest eligibility Level 1. Even though they were in the lightest level, over half were 80 and over. This could be the main reason why 27.8% had declined one and a half year later. The proportion of decline from IADL disability only to ADL disability was about 10 to 15% among those 75 and over [20-24].

Similar to previous studies, we found that people who had no primary caregiver, or whose primary caregiver came from outside the household, were more likely to choose home help, while those whose primary caregiver lived in the same household were more likely to go to day care [25,26]. However, even after controlling for these variables, home help service users were found to have a lower risk of functional decline than day care service users. And providing more home help services did not lead to greater decline in function. These findings contradict the assumptions of the Ministry's policy.

Before concluding that those assumptions are wrong, we should consider alternative explanations. First, could the day care users have been more frail than the home help users at the start? Although we have statistically controlled for age, there could be other factors such albumin to total protein level [27-29], and subjective measurement of health [30-32], which could have had implications on the subjects' intrinsic frailty. However, in the research design, subjects were limited to those of the lightest eligibility level, and the outcome required them to decline by three levels to Level 4. Therefore, any slight differences appearing at baseline or during the observation period were unlikely to be reflected in the results. Note also that only new users with no history of LTCI service use were included, and that since data was limited to usage claims within three months from initial eligibility certification, the possibility of a reverse cause-effect relationship (decline leading to the start of service) would be low.

Second, could the day care services that the subjects received be less focused on preventive care services than the norm? The Ministry's model programme, which added selective muscle strength training and improvement of nutrition and oral function on top of their former services, was reported to have been effective [33]. In theory, it would have been possible to widely replicate and disseminate this model. A systematic review of day care programmes revealed that sites that had comprehensive preventive care interventions achieved some success. However, in this review, only a few sites showed success [34] and whether these results could be generalized remains doubtful.

In Japan, although Kuzaya et al have reported that the use of day care services had a positive effect on lowering the mortality rate, both the intervention and the subjects differed from our study [35]. Kuzuya et al conducted their research prior to the implementation of the preventive services and their subjects spanned across all eligibility levels, among which two-thirds had ADL disability. An analysis of 20,000 day care service users in Tokyo conducted after the implementation showed that there were no differences in the functional decline as measured by eligibility levels between those who had selected muscle strength training and improvement of nutrition and oral function, and those who had not. Most day care services appeared to be still focussed on recreation and bathing services, and providing respite care to caregivers who live in the same household [36]. Thus, the fact that those receiving day care services in our study did not improve was not surprising.

Third, did providing excessive home help services lead to a greater proportion of the users to decline as the Ministry has claimed? Our analysis of time-dependent variables after controlling for the presence of caregiver and other factors showed no particular change in the risk of decline in functional status for those who used more than two hours of home help services per week compared to those who used less. This is not surprising. Looking at the substance of the programme, it is implausible that the provision of only one or two hours of home help services per week would lead to disuse syndrome.

Our study was limited to those who had been assessed to be in Level 1, and to those who had been using home help services or day care services, as this was focus of the new reform policy. Whether either service would have any effect compared to non-users remains to be explored, or for that matter, compared to those who have a similar level of disability, but who have not yet been assessed. Further research is needed. Our study was also confined to a single area which had a higher rate of home help service use and somewhat lower day care service use compared to the national average (Table 2). This is similar to other urban municipalities that tend to have relatively well-established home help service provision. Further studies should be made in rural municipalities that have the opposite traits.

## Conclusions

In the new preventive care policy for those requiring light care, the Ministry claimed money could be saved by encouraging day care services, and containing home help services. However, our study showed contrary results: home help service users were found to have a lower risk of functional decline than day care service users.

## Competing interests

The authors declare that they have no competing interests.

## Authors' contributions

TI was involved in study conception and design, entry, analysis, revision, editing and manuscript writing. NI was involved in study conception, manuscript writing, revision, editing and overall supervision. All authors have read and approved the final manuscript.

## Pre-publication history

The pre-publication history for this paper can be accessed here:

http://www.biomedcentral.com/1472-6963/10/224/prepub
